# Dynamic Acclimation to High Light in *Arabidopsis thaliana* Involves Widespread Reengineering of the Leaf Proteome

**DOI:** 10.3389/fpls.2017.01239

**Published:** 2017-07-20

**Authors:** Matthew A. E. Miller, Ronan O’Cualain, Julian Selley, David Knight, Mohd F. Karim, Simon J. Hubbard, Giles N. Johnson

**Affiliations:** ^1^School of Earth and Environmental Sciences, University of Manchester Manchester, United Kingdom; ^2^School of Biological Sciences, University of Manchester Manchester, United Kingdom

**Keywords:** photosynthesis, light acclimation, proteomics, electron transport, carbon fixation

## Abstract

Leaves of *Arabidopsis thaliana* transferred from low to high light increase their capacity for photosynthesis, a process of dynamic acclimation. A mutant, *gpt2*, lacking a chloroplast glucose-6-phosphate/phosphate translocator, is deficient in its ability to acclimate to increased light. Here, we have used a label-free proteomics approach, to perform relative quantitation of 1993 proteins from Arabidopsis wild type and *gpt2* leaves exposed to increased light. Data are available via ProteomeXchange with identifier PXD006598. Acclimation to light is shown to involve increases in electron transport and carbon metabolism but no change in the abundance of photosynthetic reaction centers. The gpt2 mutant shows a similar increase in total protein content to wild type but differences in the extent of change of certain proteins, including in the relative abundance of the cytochrome b_6_*f* complex and plastocyanin, the thylakoid ATPase and selected Benson-Calvin cycle enzymes. Changes in leaf metabolite content as plants acclimate can be explained by changes in the abundance of enzymes involved in metabolism, which were reduced in *gpt2* in some cases. Plants of *gpt2* invest more in stress-related proteins, suggesting that their reduced ability to acclimate photosynthetic capacity results in increased stress.

## Introduction

In order to maximize fitness in naturally variable conditions, plants must tolerate changes in their environment occurring on a wide range of different timescales. Light intensity is the most rapidly changing and variable abiotic factor, but is also the most important, given that it provides the energy to drive photosynthesis. The developmental effects of light on plants grown at different irradiances have been well studied; and a number of differences can be seen when low light (LL) grown leaves are compared with those developed at high light (HL). Typically, HL leaves are thicker, with more cell layers, and a higher chlorophyll (Chl) a:b ratio (Boardman, 1977; Walters and Horton, 1994; Bailey et al., 2001). These observations reflect a lower investment in light harvesting, with more proteins involved in energy capture, electron transport and the Benson-Calvin cycle, giving a higher capacity for photosynthesis (*P*_max_), compared to LL leaves (for a detailed review, see Walters, 2005). These acclimation responses serve to maximize photosynthetic efficiency under the prevailing light conditions.

While developmental acclimation represents a response to the conditions in which the leaf develops, dynamic acclimation to changing conditions can also occur in fully developed leaves (Athanasiou et al., 2010). Dynamic acclimation can involve plants altering their cell content, however, the leaf morphology is already set during development. The resultant leaf may therefore be suboptimal for a given irradiance. In rice, LL-developed leaves acclimated to HL are able to achieve the same *P*_max_ as HL grown leaves, even though they are thinner (Murchie et al., 2005). In contrast, in *Chenopodium album*, LL leaves transferred to HL are only able to increase their *P*_max_ to an intermediate level between that of LL and HL grown leaves (Oguchi et al., 2003). These studies suggest that changes in protein content can, in some cases, fully compensate for differences in leaf anatomy, but that the capacity for dynamic acclimation is species specific (Yin and Johnson, 2000). The ability to dynamically acclimate has been shown to vary amongst ecotypes of Arabidopsis (Athanasiou et al., 2010; van Rooijen et al., 2015). The presence of such variation suggests that acclimation ability is a trait of selective importance for some environments.

Previously, a chloroplast glucose 6-phosphate/phosphate transporter, GPT2, was shown to be required for dynamic acclimation of photosynthetic capacity to an increase in irradiance in Arabidopsis (ecotype Ws; Athanasiou et al., 2010). The importance of dynamic acclimation in naturally variable environments was further demonstrated, with *gpt2* knockout plants having a substantially lower seed yield than WT when grown in a natural light environment but not when grown in a controlled environment (Athanasiou et al., 2010). Given the inter- and intra-specific variability in acclimation potential, optimizing acclimation potential may offer an important route toward improving plant productivity. Understanding the molecular processes involved in acclimation will be an important step in this.

Recently, we characterized the early stages of acclimation to HL in Arabidopsis, using transcriptomics and metabolomics in WT and *gpt2* plants (Dyson et al., 2015). We observed that, although *gpt2* plants are phenotypically indistinguishable from the WT under LL conditions, they show a distinct transcriptional strategy, with elevated transcript levels for many photosynthetic genes relative to the WT. Upon exposure to HL, *gpt2* plants accumulate less starch and have elevated levels of many sugar intermediates, compared to the WT. As plants acclimate to HL, leaf metabolite content showed a range of responses, with initial changes in content induced by HL being followed by a tendency to return to control levels. The metabolic changes underlying this response were distinct between WT and *gpt2*.

Changes in metabolism and an increase in *P*_max_ upon HL acclimation suggest there will be changes in the proteome, however, transcript levels for virtually all polypeptides involved in photosynthesis are insensitive to increases in growth light (Walters, 2005; Piippo et al., 2006; Athanasiou et al., 2010; Dyson et al., 2015). This implies either that, under such conditions, increases in *P*_max_ are achieved through post-translational modifications or that protein abundance is controlled post-transcriptionally. Previous observations on selected proteins (e.g., Rubisco, cytochrome *f*) have shown that the total content of these increases upon exposure of plants to an increase in light (Yin and Johnson, 2000; Athanasiou, 2007; Athanasiou et al., 2010). Thus, we conclude that, for photosynthetic reactions at least, microarray analysis gives incomplete and, indeed, misleading information about changes protein content. To understand the responses of the leaves to increased light, we need to examine changes in the proteome.

In the past decade, proteomic techniques based on tandem liquid chromatography and mass spectrometry (LCMS) have started to allow the large scale quantitation of the proteome in a manner analogous to transcriptomics.

In this study, we have adopted a label-free proteomics technique to investigate changes in the Arabidopsis proteome resulting from dynamic acclimation to HL. Our aim was to test the hypothesis that impaired acclimation of photosynthesis in the *gpt2* mutant was due to a failure to alter the abundance of specific components of the leaf proteome. We observe increases in many proteins of central metabolism, and changes in the composition of complexes involved in photosynthesis. Results are shown to be consistent with our previous knowledge of the acclimation process in this plant. Examination of the proteome of the *gpt2* mutant shows that the inability of this to acclimate photosynthesis to HL is due to a failure to increase the abundance of specific proteins involved in electron transport and carbon fixation to WT levels.

## Materials and Methods

### Plant Growth Conditions

Plants of *Arabidopsis thaliana* accession Wassilewskija-4 and a homozygote T-DNA insertion knockout of the GPT2 gene (*gpt2*, FLAG_326E03; INRA, Versailles, France), were used in all experiments. Plants were grown for 8 weeks under an 8 h day (20°C day/16°C night) at a light intensity of 100 μmol m^-2^ s^-1^ (LL) under warm white LEDs (color temperature 2800–3200 K), and then transferred to an irradiance of 400 μmol m^-2^ s^-1^ (HL) for 7 days. All plants were harvested on Day 7 HL, controls were maintained at LL and harvested on the same day. Plants were harvested at the end of the photoperiod, with leaves identified as fully developed prior to treatment being flash frozen in liquid nitrogen directly from growth conditions.

### Photosynthesis, Chlorophyll Fluorescence and P700 Measurements

For all measurements of gas exchange, a CIRAS1 infrared gas analyser (PP Systems, Amesbury, MA, United States) was used. All measurements were made at a CO_2_ concentration of 2000 μl l^-1^ at an irradiance of 2000 μmol m^-2^ s^-1^. Measurements of chlorophyll fluorescence were performed using a PAM-101 chlorophyll fluorimeter (Heinz Walz, Effeltrich, Germany) with data recorded with a National Instruments PCI-6220 analog to digital convertor, using laboratory written software. Fluorescence parameters were calculated as described by Maxwell and Johnson (2000). P700 redox state was measured using a Walz PAM 101 in combination with an ED-P700DW-E emitter-detector unit (Heinz Walz; Hald et al., 2008). Actinic and saturating flash light was provided by a LED Engin LZ4 warm white LED (LED Engin, San Jose, CA, United States).

### Label-Free Proteomics

Frozen leaf samples were ground to a fine powder using a pestle and mortar under liquid nitrogen. For total protein content measurements, ground leaf was normalized on a fresh weight basis and Bradford reagent (Sigma–Aldrich, St. Louis, MO, United States) was used according to the manufacturer’s instructions for estimates of total leaf protein. For LCMS analysis, five replicates were used per condition, and 20 mg FW of ground leaf was added to 200 μl of 1% Rapigest (Waters, Milford, MA, United States) in 25 mM ammonium bicarbonate. Samples were then denatured at 80°C for 10 min. A 10 μl aliquot of this sample was reduced and alkylated by dithiothreitol and iodoacetamide. Samples were then digested overnight using proteomics grade trypsin (Sigma–Aldrich) at 37°C. Rapigest was removed by the addition of trifluoroacetic acid to 1% final concentration, and incubated for 2 h at 37°C, followed by 2 h at 4°C. Samples were centrifuged at 14,000 *g* for 15 min and the supernatant was desalted using POROS R3 beads.

Digested samples were analyzed by LC-MS/MS using an UltiMate^®^ 3000 Rapid Separation LC (RSLC, Dionex Corporation, Sunnyvale, CA, United States) coupled to an Orbitrap Elite mass spectrometer (Thermo Fisher Scientific, MA, United States). Peptide mixtures were separated using a gradient starting with a mixture of two solutions: 92% of 0.1% Formic acid (FA) in water and 8% of 0.1% FA in acetonitrile, increasing up to 33% FA in acetonitrile. Run time was a total of 180 min at a flow rate of 300 nL min^-1^. A 75 mm × 250 μm i.d. 1.7 μM BEH C18 analytical column (Waters) was used for separation. Peptides were selected for fragmentation automatically by data dependent analysis.

Raw data were imported into Progenesis QI (build 2.0.5556.29015; Nonlinear Dynamics, Newcastle, United Kingdom) and runs were aligned according to the default settings. Only ions with a charge state of up to +4 were considered. MS/MS data were searched against the *A. thaliana* TAIR 10 database and assigned to peptides using Mascot version 2.4.0 (Matrix Science, London, United Kingdom). A maximum of one missed cleavage (Trypsin) was permitted, with a peptide mass tolerance of 10 p.p.m. and an MS/MS tolerance of 0.5 Da. For a complete list of identified peptides, see Supplementary Data S2. Data were then re-imported into Progenesis to allow for assignment of proteins from peptide data.

Raw protein intensities were exported from Progenesis and, instead of normalizing all samples to a single run, a reference run was selected for each *treatment* based on the sample with the median total protein content for that treatment. Total protein for each sample was calculated by summing the intensities of all the quantified proteins (for further details see Supplementary Figure S2). All replicates in each condition were then normalized to the reference run for that treatment.

When a comparison of two conditions was made, a *t*-test was used, using the constraints detailed in the figure legends. When a comparison of more than two conditions was made, an ANOVA was used. Proteins were considered to have significantly changed in abundance when a *P*-value of <0.05 was reached, with a fold change of >1.2, unless otherwise stated. All data analysis was carried out in the R software package, except ANOVAs, which were performed in SPSS (IBM, Armonk, NY, United States). Exploratory hierarchical clustering (**Figure 2**) was done using Euclidean distance and the complete linkages method. For comparitive hierarchical clustering (**Figure 6**) and heatmap analysis, fold change data were calculated relative to the WT at LL and log_2_ scaled. A heatmap was then generated using the heatmap.2 package in the R software, using the default settings.

The mass spectrometry proteomics data have been deposited to the ProteomeXchange Consortium via the PRIDE (Vizcaino et al., 2016) partner repository with the dataset identifier PXD006598 and 10.6019/PXD006598.

## Results

### Acclimation to HL Increases Protein Content But Not Photosynthetic Capacity of *gpt2*

**FIGURE 1:**
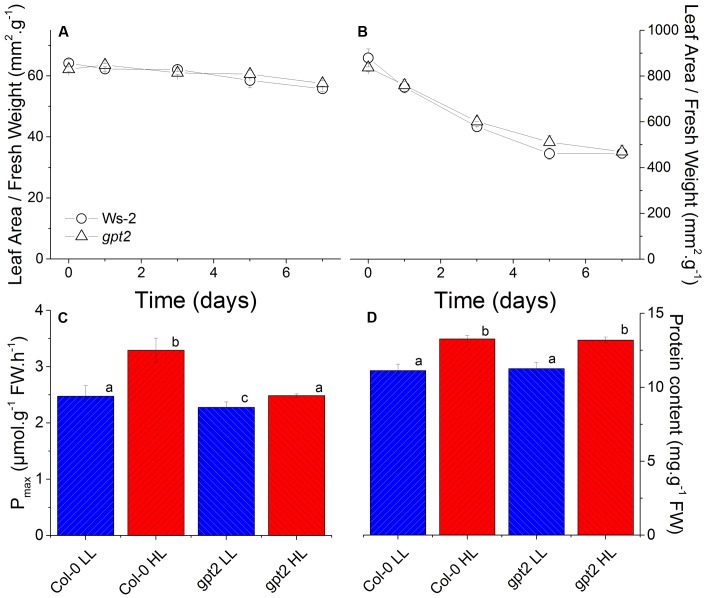
Physiological changes in WT and *gpt2* leaves following HL acclimation. Plants were grown for 8 weeks at LL (100 μmol m^-2^ s^-1^), and then transferred to HL (400 μmol m^-2^ s^-1^) for a further 7 days. Figures **(A,B)** are timecourse experiments where day 0 = LL. Leaves were harvested at the end of the photoperiod on days 0,1,3,5, and 7. Measurements of photosynthetic capacity (*P*_max_) **(C)** were made following illumination under saturating conditions (2000 μL L^-1^ CO_2_ and 2000 μmol m^-2^ s^-1^ light) for 15 min. Protein content **(D)** was determined using a Bradford assay. Different letters denote significantly different results (ANOVA, *P* < 0.05).

Plants were grown at LL (LL; 100 μmol m^-2^ s^-1^) for 8 weeks and then acclimated to high light (HL; 400 μmol m^-2^ s^-1^) for 7 days. This HL intensity was selected as, previously, this was shown to induce a non-stressing acclimation response (Yin and Johnson, 2000; Athanasiou, 2007). Leaves that were fully expanded before the HL treatment were used for analysis. This approach was taken to avoid examining developmental acclimation to HL, and to assess how leaves that developed at LL are able to alter their protein composition in response to an increase in light intensity (Athanasiou et al., 2010). Examination of leaf sections by microscopy confirmed that there were no obvious changes in leaf morphology induced by the HL treatment (Supplementary Figure S1). As plants of both genotypes acclimated to HL, the leaf area per unit fresh weight declined marginally, but significantly, by about 10% (**Figure 1A**). There was also a marked decrease in the specific leaf area, implying the accumulation of a substantial amount of dry matter in the leaves (**Figure 1B**).

Exposure of wild type plants to HL resulted in an increase in their maximum capacity for photosynthesis (*P*_max_) per unit leaf weight, from 2.5 to 3.3 μmol CO_2_ g^-1^ hr^-1^ (**Figure 1C**), consistent with previous results (Athanasiou et al., 2010). In contrast, plants lacking the glucose-6-phosphate translocator GPT2 were only able to reach a *P*_max_ of 2.5 in response to HL, similar to WT at LL (**Figure 1C**). Both WT and *gpt2* leaves did, however, contain approximately 20% higher total protein content per unit fresh weight after HL acclimation (**Figure 1D**).

### Label-Free Proteomics Provides Extremely Deep Coverage of the Proteome

To define changes occurring in the leaf proteome in response to HL, we adopted a label-free proteomic approach. In order to maximize the reproducibility and breadth of coverage of the proteome, various methods of protein purification and separation were explored. The best results were found with a simple ‘in-solution’ digestion protocol. Leaf proteins were solubilised in a buffer containing Rapigest (Waters, Milford, MA, United States), before being digested with trypsin, desalted and analyzed using LCMS. This avoided the additional steps involved in gel-based fractionation or isobaric labeling and allowed the entire leaf proteome to be analyzed in a single MS run. A total of 3514 proteins were reproducibly identified on the basis of 36380 unique peptide IDs (see Supplementary Data S1, S2). We applied a strict filter to the dataset, so that proteins were only quantified when three or more non-redundant constituent peptides were reproducibly identified. With this filter, 1993 proteins per run could be quantified, which were involved in diverse metabolic processes (for a complete list of quantified proteins, see Supplementary Data S1).

One of the challenges of quantifying proteomes is to determine the basis for normalizing between samples. It is a common practice to assume that all samples have the same protein content per unit of material. Using the Progenesis default normalization method, it was found that in the WT, of the 1993 quantified proteins, 251 increased and 369 decreased in abundance. Proteins were considered to have significantly changed in abundance when a *p*-value of <0.05 or lower was reached, with a minimum fold change of 1.2, a level widely adopted in proteomics experiments (Nissom et al., 2006; Keenan et al., 2009; Serang et al., 2013; Zhang et al., 2016). In the present case, however, using such a normalization arguably gives misleading results. For example, on this basis, we would have to conclude that investment in electron transport and carbon fixation were unchanged or declined in leaves acclimated to HL. This does not reflect the increase in *P*_max_ (**Figure 1C**) or observations from western blotting of RBCS, which showed an increase per unit leaf weight in WT plants (data not shown). Furthermore, this normalization method showed no increase in total protein after acclimation to HL (calculated by summing all protein quantified proteins), in contrast to results from the Bradford assay, which showed a 1.2-fold increase (**Figure 1D**).

An assumption of the Progenesis default normalization is that the majority of detected features (peptides) do not change in abundance between samples, and thus these features can be used for normalization. A reference sample is then selected and all samples are normalized according to the intensity of the selected normalization vectors. In essence, this approach is an automated method of selecting a number of housekeeping peptide ions that can be used for normalization. Under our experimental conditions, however, this assumption was violated, as the increase in total protein content under HL (**Figure 1D**) resulted in an increase in many of the detected features, leading to the erroneous results as described in the previous paragraph. Therefore, a normalization method based upon the median total protein content in each experimental group was used (for further details, see Materials and Methods and Supplementary Figure S2). After normalization, a large proportion of the proteome showed a significant increase in abundance after acclimation, consistent with the increase in total leaf protein (**Figure 1D**). For WT and *gpt2* plants, 1284 and 1119 proteins were significantly increased in abundance, respectively, with only 14 proteins being significantly decreased for both genotypes. 831 proteins were increased in both WT and *gpt2*.

Amongst the quantified proteins, extremely good coverage of central carbon metabolism was achieved (for further details see Supplementary Figure S3). This included quantitation of components of every major protein complex of the photosynthetic electron transport chain and every enzymatic step of the Benson-Calvin cycle. Furthermore, we were able to quantify the majority of enzymes from sucrose and starch metabolism, glycolysis and respiration. Many proteins involved in other key metabolic pathways were also quantified, including amino acid, lipid, tetrapyrrole and antioxidant metabolism. The majority of identified proteins were easily solubilised or abundant enzymes, while there was an under representation of membrane proteins.

**FIGURE 2:**
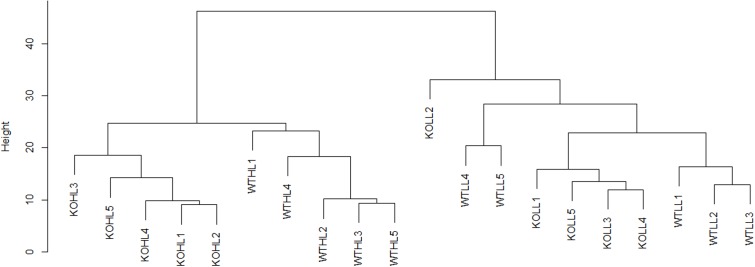
Hierarchical clustering analysis of WT and *gpt2* plants exposed to HL. A dendrogram was constructed using log_2_ scaled intensities for the 1993 quantified proteins, using the Euclidean distance and the complete linkages method. Each of the five biological replicates were plotted for every condition. Clear separation can be seen between LL and HL cluster, and also between WT and *gpt2* at HL.

For initial data analysis, hierarchical clustering was performed (**Figure 2**). LL samples were clearly separated from HL samples, indicating that the light treatment caused a change in the proteome of both WT and *gpt2* plants and that the effect of the treatment was stronger than the effect of the genotype. When the LL cluster is examined, there is only poor discrimination between WT and *gpt2*, suggesting the LL proteomes are rather similar between genotypes. However, following acclimation to HL, the genotypes separate into distinct clusters, indicating different responses are occurring.

### The Thylakoid Proteomes of Wild Type and *gpt2* Plants Respond Differently to High Light

**FIGURE 3:**
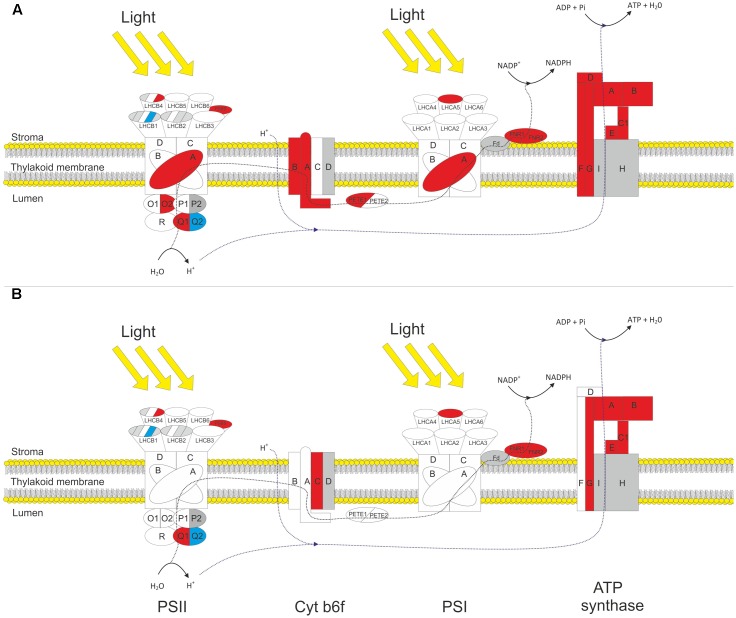
Response of thylakoid protein complexes to HL in WT and *gpt2* plants. Diagram showing significantly changing protein subunits involved in the photosynthetic electron transport chain for WT **(A)** and *gpt2* **(B)** plants. Subunits not quantified are colored gray, detected subunits that do not significantly change in abundance are colored white, increased subunits colored red and decreased subunits blue. Protein subunits were considered to have significantly changed in abundance when a minimum threshold of 1.2-fold change, with a *p-*value of <0.05 was achieved.

The proteomic analysis provided good coverage of the protein complexes of the thylakoid membrane. **Figure 3** summarizes how different gene products associated with the light reactions respond to HL in both WT and *gpt2* plants. All proteins involved in core PSII function (PSBA-F) were detected, and most PSI core proteins (PSAA-F). Even highly homologous isoforms of the same polypeptide could, in some cases, be discriminated (e.g., PSBO1 and PSBO2, which share 95% amino acid homology; Dasgupta et al., 2005). The analysis also provided good coverage of the rest of photosynthetic electron transfer, including quantification of every subunit from the CF1 domain of the chloroplast ATP synthase, both plastocyanin and photosynthetic FNR isoforms, and most, but not all, subunits of the cytochrome (Cyt) b_6_*f* complex.

Amongst the proteins involved in light capture, similar trends were seen in the two genotypes. The majority of detected PSII antenna proteins showed no significant change in response to HL, however, specific proteins did change. LHCB 4.3 (AT2G40100) showed a more than threefold increase in both genotypes, while LHCB 1.4 (AT2G34430) was decreased by 50% in WT plants but only 30% in gpt2 plants. There was also a significant increase in LHCA 5 (AT1G45474) in both genotypes. Thus, these results suggest a reorganization of the light harvesting proteins is occurring in both genotypes.

In the WT, there were also increases of around 50% in the protein NPQ4 (PSBS; AT1G44575), which is required for non-photochemical quenching. A similar increase was also seen in NPQ1 (violaxanthin deepoxidase). In *gpt2* plants, NPQ4 behaved in a similar manner to the WT, however, NPQ1 was not significantly altered in response to HL.

Although a large proportion of the proteome was increased in absolute terms in response to HL, the majority of PSII core proteins showed no change in abundance (including PSBB-D). PSBA (ATCG00020) did show a small but significant (1.25-fold) increase in WT plants. In *gpt2*, PSBA was detected at a higher level than WT at LL but did not change significantly in response to HL. Assuming that these proteins are only functional as part of a complex, it is unlikely that an increase in a single subunit reflects a change in the abundance of the reaction center complex. It was possible to detect isoforms of all proteins of the oxygen evolving complex of PSII (OEC), with both isoforms being quantified for PSBO and PSBQ. Amongst the quantified proteins, a change in the relative abundance of the different isoforms was seen. For example, there was an increase in PSBQ1 (AT4G21280), accompanied by a similar decrease in PSBQ2 (AT4G05180), suggesting that at HL, the Q1 isoform is favored. Similarly, for PSBO, we saw no increase in the O1 (AT5G66570) isoform, yet an increase in the O2 (AT3G50820) isoform. Both PSBP isoforms were detected, however, due to the high sequence homology between these isoforms, there were too few unique peptides to allow quantification of PSBP2. PSBP1 and PSBR were detected but showed no change in abundance. Overall, these results indicate a changing composition of the OEC upon HL acclimation. There is no clear evidence that total PSII abundance changes upon acclimation to HL in either genotype.

The only apparently increased protein detected in the PSI core complex was PSAA (ATCG00350) in the WT, by 30%.

When proteins involved in the photosynthetic electron transport chain (PETC) were examined, a clearer pattern of increased abundance was apparent. In WT there were significant increases in 2 of the 3 Cyt b_6_*f* subunits, 1 plastocyanin isoform, both FNR isoforms, and all detected ATP synthase subunits. Although showing a similar trend to the WT, *gpt2* plants increased less of the Cyt b_6_*f* complex, with only one detected subunit being considered *significantly* altered, and no change in plastocyanin content being observed.

**FIGURE 4:**
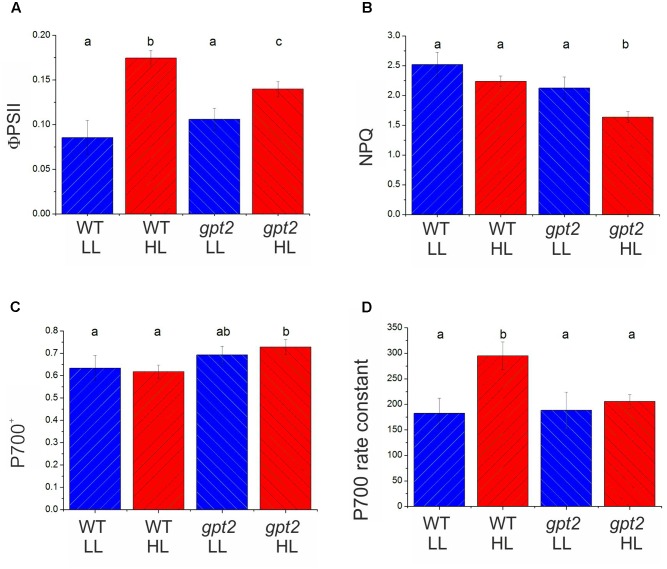
Chlorophyll fluorescence and PSI absorbance parameters of WT and *gpt2* plants upon HL acclimation. Plants were grown for 8 weeks at LL (100 μmol m^-2^ s^-1^), and then transferred to HL (400 μmol m^-2^ s^-1^) for a further 7 days. Chlorophyll fluorescence measurements were used to calculate ΦPSII **(A)** and NPQ **(B)** following 30 min illumination at 2000 μmol m^-2^ s^-1^ and 2000 μL L^-1^ CO_2_. PSI redox state **(C)**, and the rate constant for P700 re-reduction following darkening **(D)** were estimated by following changes in near infra-red absorbance (830–870 nm). Error bars = standard error, different letters denote significantly different data (ANOVA, *p* < 0.05).

In order to determine whether the above conclusions, from proteomic analysis of electron transport proteins are consistent with behavior of the photosynthetic apparatus, chlorophyll fluorescence analysis was performed (**Figure 4**). The parameter ΦPSII gives an estimation of the proportion of absorbed light energy that is used for photochemistry (Genty et al., 1989). Under saturating CO_2_ and light conditions in WT plants, ΦPSII increased by around 50% at HL (**Figure 4A**). This compares with an increase of approximately 30% in *P*_max_, measured under the same conditions (**Figure 1C**). In *gpt2* plants, ΦPSII increased to a lesser extent than in the WT. NPQ was found to not change significantly in response to HL in the WT, however, in *gpt2*, a significant *reduction* in NPQ was observed in HL acclimated leaves (**Figure 4B**).

Proteomic data suggest that changes are occurring in the relative abundance of electron transport proteins, with photosystems being unaltered whilst proteins responsible for intersystem electron transport tending to increase. This is predicted to affect the flux through the electron transport chain. Measurements of the reduction kinetics of PSI were conducted by measuring the oxidation state of the primary electron donor, P700 (Harbinson and Hedley, 1989; Ott et al., 1999). At 2000 μmol m^-2^ s^-1^ light, the degree of oxidation of P700 did not vary significantly between plants maintained at LL and those transferred to HL in either genotype (**Figure 4C**). P700 was more oxidized in the mutant, however, in both treatments. P700 re-reduction following a transition to darkness was fitted with a single exponential decay curve, yielding a pseudo-first order rate constant indicative of the conductance of the electron transport chain (Golding and Johnson, 2003). In WT plants, the rate constant in HL plants was 60% higher than in LL plants (**Figure 4D**), indicating an increased conductance of the PETC, consistent with the increase in the relative abundance of electron transport components. In *gpt2* plants, there was no significant increase in the rate constant in HL-acclimated plants. Taken together these measurements indicate that, in the WT, the overall flux per PSI reaction center was greater in HL plants, whilst in the *gpt2* mutant, no acclimation of electron transport occurred. Combined with ΦPSII data (**Figure 4A**), these data indicate that HL acclimated WT plants have a greater relative capacity for linear electron flow than *gpt2*, consistent with our interpretation of the proteomic data. The Cyt b_6_*f* complex is the earliest part of the PETC where differences are apparent between WT and *gpt2* plants at HL.

### Acclimation to High Light Requires Increases in Enzymes of Central Carbon Metabolism

**FIGURE 5:**
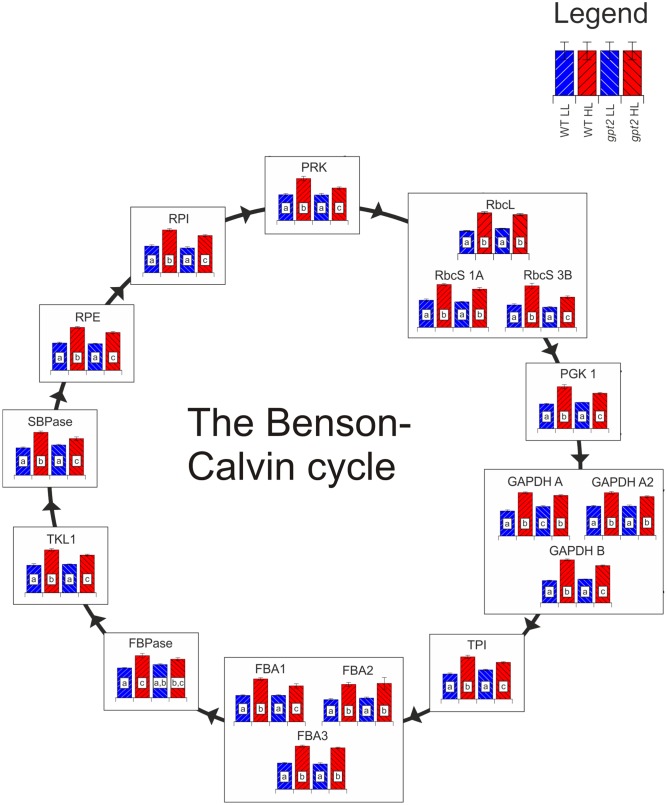
The response of the Benson-Calvin cycle to HL acclimation in WT and *gpt2.* Quantified proteins are diagrammatically represented in this figure, with each column representing a treatment according to the legend in the top right of the figure. Significantly different results are denoted by different letters within each column (ANOVA, *p* < 0.05). When more than one protein isoform was quantified, all are shown. Abbreviations: RbcL, Ribulose bisphosphate carboxylase large subunit; RbcS, Ribulose bisphosphate carboxylase small subunit; PGK1, phosphoglycerate kinase 1; GAPDH, glyceraldehyde 3-phosphate dehydrogenase; TPI, triose phosphate isomerase; FBA, fructose-bisphosphate aldolase; FBPase, Fructose 1,6-bisphosphatase; TKL, transketolase; SBPase, sedoheptulose-1,7-bisphosphatase; RPE, ribulose 5-phosphate epimerase; RPI, ribose 5-phosphate isomerase; PRK, phosphoribulokinase. In the case of TPI, too few unique peptides were quantified to allow individual isoform discrimination, so shared peptides between TPI1 and TPI2 were used to quantify total TPI.

All of the detected Benson-Calvin cycle enzymes were increased in the WT at HL, by on average 50% (**Figure 5**), consistent with the increased capacity for carbon fixation (**Figure 1C**). While the majority of enzymes were also increased in *gpt2*, this increase relative to the control conditions was lower for many enzymes, in the range of 10–30%. Many of the more abundant Benson-Calvin cycle enzymes were quantified using nine or more peptides, allowing extremely robust quantitation, allowing even slight differences in abundance to be resolved. Surprisingly, the only enzyme that did not show differing abundance between the genotypes at HL was Rubisco, with a 75% increase in the large subunit (RBCL; ATCG00490) for both genotypes. For the Rubisco small subunit (RBCS), several different isoforms could be detected. When common peptides for all RBCS isoforms were used to calculate total RBCS abundance, there was no difference between WT and *gpt2*. However, it was also possible to detect enough unique peptides to quantify RBCS 1A (AT1G67090) and 3B (AT5G38410) individually. At HL, there was a difference in the RBCS isoform balance, with a similar increase in RBCS 1A in both WT and *gpt2*, but an increase RBCS 3B to a significantly higher level in the WT. Even though *gpt2* plants are affected in their ability to increase their capacity for carbon fixation upon HL, overall Rubisco abundance increases in a manner similar to the WT, indicating that the abundance of this enzyme is not sufficient to explain the difference in *P*_max_. The abundance of other Benson-Calvin cycle enzymes, in particular sedoheptulose 1,7-bisphosphatase (SBPase; AT3G55800) and fructose 1,6-bisphosphate aldolase (FBA) have previously been suggested to be important in determining *P*_max_ (Zhu et al., 2007). We were able to quantify SBPase and all three chloroplast localized FBA isoforms (FBA 1–3; Lu et al., 2012). Both SBPase and FBA1 (AT2G21330) were increased to a significantly lower level at HL in *gpt2* compared to the WT.

In the WT, nearly all enzymes of starch and sucrose metabolism were increased in abundance, suggesting an increased capacity for flux through these pathways (see Supplementary Figure S4). A similar pattern of increased abundance was observed in *gpt2* plants, however, as with the Benson-Calvin cycle, a number of enzymes were detected at a lower level at HL, relative to the WT. There were 13% lower levels of the starch synthesis enzymes STARCH SYNTHASE 1 (SS1; AT5G24300) and ADP-GLUCOSE PYROPHOSPHORYLASE (AGPase; AT5G19220) at HL. *gpt2* plants also contained on average 24% lower levels of enzymes associated with starch degradation, including 2 β-amylases (BAM3; AT4G17090 and BAM6; AT2G32290), and DISPROPORTIONATING ENZYME (DPE1; AT5G64860). Overall, lower levels of starch metabolism enzymes in *gpt2* plants, compared to WT at HL, suggests a lower capacity for this pathway.

Sucrose metabolism exhibits a similar pattern of increased abundance to starch metabolism under HL, as all of the detected enzymes in sucrose synthesis are increased in WT (Supplementary Figure S4). The same is true of *gpt2* plants, with the exception of selected enzymes, including SUCROSE PHOSPHATE SYNTHASE 2F (SPS2F; AT5G11110). The sucrose degrading enzymes SUCROSE SYNTHASE 1 and 4 (SUS1; AT5G20830 and SUS4; AT3G43190) are also increased in WT plants and not in *gpt2* plants at HL. It should, however, be noted that the levels of SUS1 and 4 are higher in *gpt2* plants at LL than in the WT. Two key regulatory enzymes, HEXOKINASE 1 (HXK1; AT4G29130) and cytosolic fructose bisphosphatase (cFBPase; AT1G43670) were increased in both genotypes, but to significantly higher levels in WT than in *gpt2* at HL. HXK1 acts both catalytically in sugar metabolism but also as a sensor of glucose status (Moore et al., 2003; Cho et al., 2006), while cFBPase acts in sucrose synthesis, and also as a fructose sensor (Cho and Yoo, 2011).

The majority of enzymes involved in glycolysis and the TCA cycle were increased in both genotypes at HL (Supplementary Figure S3). There were very few differences between the WT and *gpt2* mutant in the enzymes of glycolysis at HL, and a similar pattern was also seen in the TCA cycle. Generally, glycolysis and the TCA cycle were covered well in this analysis, with at least one protein quantified for every enzymatic step of the cycle. The mitochondrial electron transport chain was less well covered; however, we were still able to quantify many of the complexes based on at least one protein. When respiration is considered as a whole, there is an increase in both WT and *gpt2* upon HL.

### Cluster Analysis Reveals Metabolic Differences between WT and *gpt2* Plants, Even at Low Light

**FIGURE 6:**
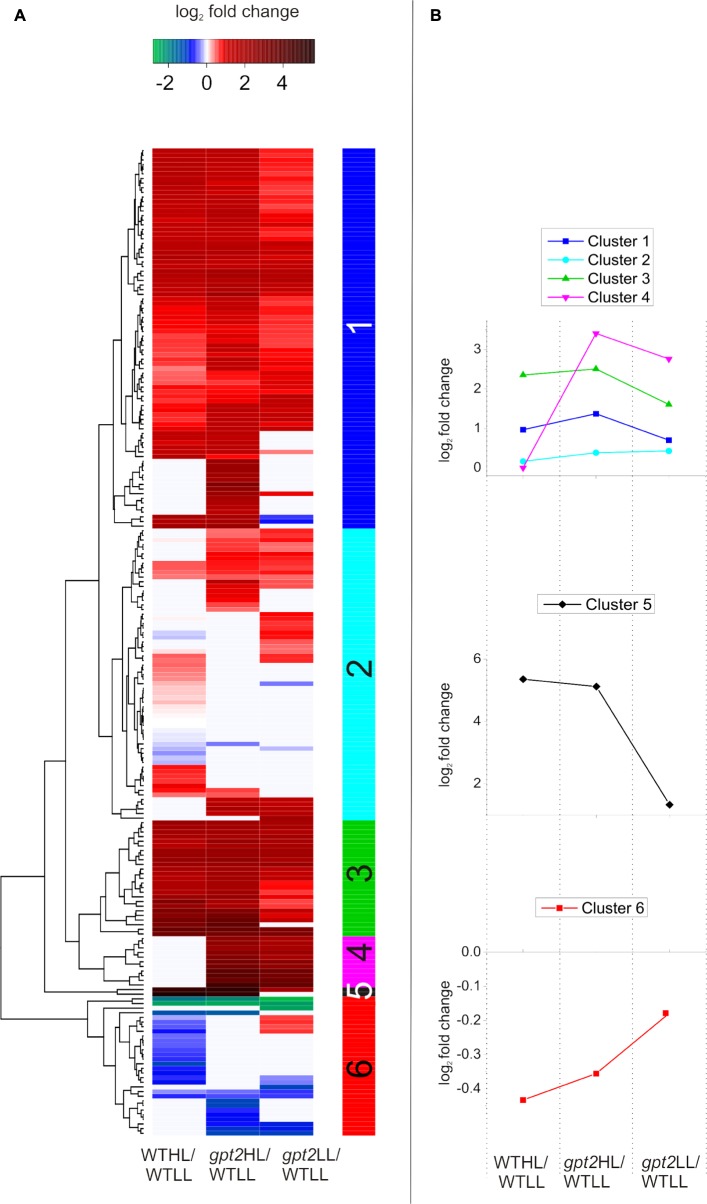
Cluster analysis of protein abundance for proteins with altered accumulation between WT and *gpt2* plants upon HL acclimation. **(A)** Displays a heatmap, with **(B)** showing the behavior of each cluster identified, as defined by the vertical colored bar. Data were initially filtered to only include proteins which were differentially expressed between the WT and *gpt2* at LL, HL, or both conditions (*T*-test, fold change >1.5, *p* < 0.05). Data were then log_2_ scaled and fold changes calculated relative to the WT at LL (WTLL), and a heatmap was constructed. Proteins that did not significantly change in each comparison are colored white.

In order to further investigate the differences between the WT and *gpt2* acclimation responses, taking a less targeted approach, cluster analysis was performed using a subset of proteins which were most differentially expressed (*p* < 0.05, greater than 1.5-fold difference in abundance) between the genotypes at either LL, HL, or both (**Figure 6**). Relative abundance was expressed relative to the WT at LL, so that each comparison is normalized to the same reference. Six clusters were defined, which display differing behavior. For a full list of proteins belonging to each cluster, see Supplementary Data S3. Proteins in Clusters 1 and 3 could be generally characterized as having elevated abundance at LL in *gpt2* but a similar pattern of increase in both genotypes at HL. A number of proteins involved in stress responses were contained within this group, including PATHOGENESIS-RELATED GENE 5 (PR5; AT1G75040), GLUTATHIONE S-TRANSFERASE 6 (GST6; AT1G02930), and HEAT SHOCK PROTEIN 60-2 (HSP 60-2; AT2G33210). GST6 was particularly strongly increased – 9-fold and 11-fold in *gpt2* and WT, respectively. Proteins involved in the isoprenoid pigment biosynthesis pathway were also found in this cluster. Overall, this suggests that, although HL induces stress response proteins in both WT and *gpt2* plants, these tend to be expressed more highly in *gpt2* at both LL and HL, relative to the WT.

Cluster 2 contained proteins with a general trend toward being expressed more highly in *gpt2*. N-MYRISTOYL TRANSFERASE-1 (NMT1; AT5G57020), regulates protein activity by lipid modification of the N-terminus (*N*-myristoylation) of a small number of proteins, including the global metabolic regulator, SUCROSE NON-FERMENTING RELATED KINASE 1 (SnRK1; AT3G29160; Pierre et al., 2007). This protein doubled in abundance in response to HL in the WT, while it remained unchanged in *gpt2*. Also in this group was PLASTID MOVEMENT IMPAIRED 2 (PMI2; AT1G66840), which was unchanged in abundance in the WT, but constitutively increased in *gpt2*. PMI2 is involved in chloroplast photo-relocation as part of the HL avoidance strategy, suggesting this response in enhanced in *gpt2*.

Cluster 4 contained proteins which showed perhaps the most striking differences between WT and *gpt2*, with no change in response to HL in the WT, but abundance in *gpt2* of on average 7-fold higher at LL, and 11-fold at HL, relative to the WT. This group included PLASTID MOVEMENT IMPAIRED 15 (PMI15; AT5G38150). PMI2 was also constitutively increased in *gpt2*, and has been shown to physically interact with PMI15 (Kodama et al., 2010) to mediate chloroplast photorelocation, suggesting an enhanced HL avoidance response in *gpt2*. L-GALACTONO-1,4-LACTONE DEHYDROGENASE (GLDH; AT3G47930), which catalyzes the final step of ascorbate biosynthesis was also found within this group, as was MYO-INOSITOL-1-PHOSPHATE SYNTHASE 1 (MIPS1; AT4G39800). Finally, SnRK1.2 was also found within Cluster 4, and was increased four and eightfold in *gpt2* LL and *gpt2* HL, respectively, relative to LL WT plants.

Only two proteins were found in Cluster 5, and these were substantially increased at HL, with both being involved in anthocyanin biosynthesis. CHALCONE ISOMERASE LIKE (CHIL; AT5G05270), was increased 13-fold in the WT, but 36-fold in *gpt2*. UDP-GLUCOSE:CYANIDIN 5-*O*-GLUCOSYLTRANSFERASE (5GT; AT4G14090), an anthocyanin glycosylating enzyme (Tohge et al., 2005), was expressed to a 4-fold higher level at LL in *gpt2* relative to WT, but increased by around 35-fold in both genotypes at HL relative to the WT at LL.

When proteins considered within the cluster analysis are compared as a whole, some trends can be seen. Across all clusters, there are many proteins involved protein synthesis regulation, such as ribosomal proteins, tRNAs and chaperones. Regulatory proteins, such as tetratricopeptide repeat proteins and translation initiation factor proteins were also abundant (for a full list, see Supplementary Data S3). This data suggests a differential regulation of protein composition between WT and *gpt2*, and that post-transcriptional regulation may be important in explaining differences between WT and *gpt2*. Many proteins involved in stress responses, antioxidant synthesis and HL avoidance were expressed more highly in *gpt2* at LL compared to the WT, suggesting GPT2 is important under control conditions. Furthermore, while some of these stress-related proteins were induced in both WT and *gpt2* at HL, generally, the induction was stronger in *gpt2*.

## Discussion

Plants in natural environments have to cope with climates which change on timescales ranging from seconds to months. There is growing evidence that climate fluctuations are a major determinant of crop yield. To cope with this plants possess, to a greater or lesser extent the ability to acclimate to the environment, i.e., to change the composition of their tissues, in terms of proteins, lipids, metabolites etc, to optimize their growth to suit the conditions experienced. In an earlier study, we showed that the ability of plants to acclimate in an increase in light was an important determinant of plant fitness (Athanasiou et al., 2010). Our understanding of the sensing and signaling pathways involved, and of the detailed changes occurring, when plants acclimate remain at a basic level, however. If we are to produce crops with increased tolerance of environmental stress to meet growing food demands, this lack of understanding needs to be addressed.

In recent years, transcriptomics and mass spectrometry based techniques for measuring metabolites, combined with the modeling approaches of systems biology, have allowed us to aspire to gain a complete understanding of the functioning of organisms at a molecular level (Katagiri, 2003; Yuan et al., 2008; Weckwerth, 2011; Srivastava et al., 2013). There is, however, growing recognition that this information is insufficient to understand all cellular processes, with studies showing non-correlations between transcript and functional protein abundance (Baginsky et al., 2005; Piippo et al., 2006). This includes specifically our changes associated with photosynthesis and associated metabolism. Our own experience, as well as data from others, is that microarray data for example do not provide good information as to changes in photosynthetic capacity (Walters, 2005; Piippo et al., 2006; Athanasiou et al., 2010). This requires an ability to measure changes in the proteome directly. With recent developments in mass spectrometry and analysis, it is now possible quantify a large proportion of the proteome using a label-free procedure (Zybailov et al., 2009; Friso et al., 2010; Majeran et al., 2010; Vanderschuren et al., 2014). Here, we have used label-free proteomics to assess dynamic changes in the proteome of fully developed leaves in response to changes in light. Our results provide detailed information that is consistent with our previous understanding of the experimental system, giving us confidence in the quantitation, but also provide insights into the process of acclimation that were missing from microarray studies. To our knowledge, we have achieved the highest coverage of the leaf proteome from a single sample without fractionation, reproducibly identifying nearly 3500 proteins, around 2000 of which could be quantified based on 3 or more non-redundant peptides. In some cases, the ability to identify protein isoforms surpassed that of microarrays – for example, the Arabidopsis ATH1 Genome Array (Affymetrix, Santa Clara, CA, United States) does not discriminate RbcS isoforms, however, this was to some extent possible in our analysis (**Figure 5**).

While we were able to obtain deep and accurate coverage of the proteome, issues around data processing and normalization have been highlighted. It is commonly assumed that total protein content is unaffected by experimental treatments and thus can be used for normalization. In the leaf, a single protein, Rubisco, represents a substantial proportion of total protein and any change in Rubisco will therefore cause a change in total protein content per unit leaf weight. Therefore, normalizing to the total protein may give misleading results. We were able to maintain the differences in protein content between treatments by normalizing across the five replicates within each experimental group separately. Using this approach, we are able to define key steps in photosynthesis that respond to changes in growth irradiance and to identify specific reactions that contribute to the acclimation-deficient phenotype we have previously observed in the *gpt2* mutant.

Based on our analysis, we conclude that the increase in *P*_max_ seen in response to a transition from low to HL is associated with an increase in the capacity of electron transport and ATP production with there being little evidence for changes in the abundance of photosynthetic reaction centers. In turn, enzymes of the Benson-Calvin cycle are increased, giving a greater capacity for carbon metabolism. These data are consistent with previous studies which demonstrated changes in selected photosynthetic proteins at HL (Yin and Johnson, 2000; Bailey et al., 2001; Walters, 2005; Piippo et al., 2006; Athanasiou, 2007; Ballottari et al., 2007; Schmitz et al., 2012; Schoettler and Toth, 2014). For example, we previously used SDS-PAGE and Western blot analysis to show that dynamic acclimation to increased light similar to that used here resulted in an increase in cytochrome f and Rubisco (Yin and Johnson, 2000). Similar observations were made for developmental acclimation to light (Bailey et al., 2001).

Examination of the protein complexes in the thylakoid membrane throws into light some of the challenges facing proteomics. Although membrane proteins were underrepresented in our data set, we nevertheless were able to get excellent coverage of this abundant proteome. For most polypeptides involved, it is safe to assume that relative abundance within a complex is fixed. Therefore, where individual subunits are seen to change significantly, these changes must be viewed with caution. In the case of the core reaction centers, a single polypeptide (PSBA and PSAA) was seen to change significantly in each case. This may be because any changes occurring in photosystem abundance are just at the limits of sensitivity of our method. In view of this, to minimize the risk of Type I errors, we have taken a consensus approach to analyzing our data – generally the more subunits that are seen to change, the more confidence we have in our conclusions. On this basis, we conclude that neither PSII nor PSI change substantially during HL acclimation. We do, however, have stronger evidence indicating a change in the relative abundance of subunits of the OEC, consistent with previous studies suggesting that these may be functionally different (Ifuku et al., 2005; Murakami et al., 2005; Lundin et al., 2007; Allahverdiyeva et al., 2009; Yi et al., 2009).

Similar changes in the relative abundance of light harvesting complexes could also be seen. We were not able to quantify all LHC proteins expected to be present (**Figure 3**), with in particular the major pools of LHCB 1 and 2 being poorly resolved. Nevertheless, we did see a marked and significant decrease in the abundance of LHCB 1.4 which shows low sequence homology to other members of the LHCB1 family (Jansson, 1999), consistent with changes in leaf chlorophyll a:b content seen under our experimental conditions (Athanasiou et al., 2010). At the same time, a number of LHC proteins previously denoted as ‘rarely expressed,’ due to their low transcript levels (Klimmek et al., 2006), were detected, including LHCB 4.3 (AT2G40100), LHCA 5 (AT1G45474) and LHCA 6 (AT1G19150). LHCB 4.3 and LHCA 5 were substantially *increased* in HL, consistent with the suggestion that these proteins may play photoprotective roles at HL (de Bianchi et al., 2011; Mishra et al., 2012; Floris et al., 2013). This is not reflected as an increase in the extent of NPQ, however, the significant *decrease* in NPQ in the *gpt2* mutant at HL is consistent with a failure of these plants to correctly adjust to HL. Previous work has highlighted that for example overexpression of PSBS (AT1G44575) can give rise to an increase in NPQ capacity (e.g., Li et al., 2002). Although we saw an increase in PSBS content in both plant lines, neither showed increased NPQ capacity and indeed the capacity in *gpt2* was slightly reduced. This reflects the complexity of factors underlying protective quenching, with pH gradient and zeaxanthin content both being important. The increase in PSBS may reflect an increase in the sensitivity of NPQ to the environment and compensate for a decrease in other factors following acclimation. It may be that the dynamics of NPQ formation and relaxation are more important to overall plant fitness in particular environments (Kromdijk et al., 2016) and the differential responses in violaxanthin deepoxidase may therefore be an important component of the reduced fitness of *gpt2* plants under fluctuating conditions.

In contrast to the photosystems, nearly all detected proteins involved in electron transport were increased at HL in the WT, as were subunits of ATPase and FNR. This is consistent with an increase in the relative capacity for electron flow between the photosystems, which is also seen in measurements of PSII and PSI electron flow (increase in ΦPSII and P700 rate constant; **Figure 4**). In *gpt2*, there is a significantly smaller increase in ΦPSII and no change in P700 rate constant, consistent with a smaller increase in electron transport capacity. Consistent with this, in *gpt2* plants fewer Cyt b_6_*f*, plastocyanin, ATP synthase and FNR peptides were increased significantly at HL. Various studies have shown the Cyt b_6_*f* complex, ATP synthase and FNR to be important in determining capacity for both electron transport and carbon fixation (Price et al., 1998; Ruuska et al., 2000; Yamori et al., 2011; Lintala et al., 2012). These data suggest that the ability of *gpt2* plants to acclimate the PETC to HL is impaired compared to the WT.

Benson-Calvin cycle enzymes were significantly increased at HL in both WT and *gpt2* plants though with protein content in the latter being significantly lower. The exception to this was Rubisco, with the total pool of RBCS and RBCL being increased equally in both the WT and *gpt2*, as was Rubisco activase. This suggests firstly that Rubisco content does not limit photosynthetic capacity in *gpt2* and secondly that it may be regulated via a different signaling pathway to other Benson-Calvin cycle enzymes. This is perhaps not surprising, since Rubisco is the only Benson-Calvin cycle enzyme containing a chloroplast encoded subunit (RBCL). It may be that accumulation of RBCL is the primary regulated process, with the accumulation of RBCS and Rubisco activase being matched to the abundance of RBCL via a retrograde signaling pathway. This suggests that multiple independent signals are required for acclimation, and a change in the redox state of the PETC alone is not sufficient alone to induce acclimation. We observed a higher abundance of SBPase and FBA1 at HL in the WT relative to *gpt2*, consistent with the suggestion that the abundance of these enzymes can be important determinants of photosynthetic capacity (Olcer et al., 2001; Zhu et al., 2007; Uematsu et al., 2012).

Considering the wider reactions of carbon metabolism, there is in general a similar response in WT and *gpt2*, consistent with the capacity for individual reactions generally increasing in response to an increased supply of substrates. We saw previously that leaf metabolite pools, after an initial disturbance (increase or decrease) upon transfer to HL, tend to return toward LL levels as acclimation proceeds. The increase in starch metabolic enzymes in some cases was smaller in *gpt2* than WT, however, again consistent with metabolic data (Supplementary Figure S4). The increase seen in the concentration of mitochondrial proteins, including enzymes required for the TCA cycle and respiratory electron transport are consistent with the increase in actual rates of respiration we saw previously (Dyson et al., 2015) and highlight the tight links between photosynthesis and respiration.

Accumulation of sucrose is less easily interpreted as the steady state leaf content of this does not reflect the rate of flux into sucrose, which is continually exported from the leaf (Winter and Huber, 2000). Sucrose phosphate synthase (SPS) is a key regulatory enzyme of sucrose synthesis (Huber and Huber, 1996; Dyson et al., 2015). Of the three detected SPS isoforms, all were increased in the WT while only two were increased in *gpt2*, with SPSA2 (AT5G11110) failing to respond to HL. SPSA2 has previously been suggested to play a role in cold acclimation (Guy et al., 1992; Lehmann et al., 2008), and data here suggest that SPSA2 may be also be responsive to HL acclimation. This supports the suggestion by Volkert et al. (2014) that SPSA2 is regulated by different signals to the other isoforms, with this signal lacking in *gpt2*. In addition to lower HL levels of SPSA2, *gpt2* plants also possess significantly lower levels of sucrose-phosphate phosphatase (SPP), which catalyzes the final step of sucrose synthesis. These data suggest that, even though the HL steady state levels of sucrose are the same between WT and *gpt2*, flux through the sucrose pool is probably higher in the WT, consistent with the higher rate of photosynthesis (Dyson et al., 2015).

Given the function of GPT2 as a sugar phosphate/phosphate translocator, it is likely that its activity mediates changes in metabolism by altering the partitioning of sugar phosphates between chloroplast and cytosol. There is already considerable evidence linking various sugar phosphates to cell signaling (Rolland et al., 2006; Haeusler et al., 2014). The observation that various known regulatory proteins (HXK1, cFBPase, SnRK1.2) respond differently to HL in the two genotypes suggests that aspects of acclimation may be mediated via these. SnRK1 activity is modulated by a number of different signals (Nunes et al., 2013a,b; Crozet et al., 2014), and although showing an upward trend in response to HL, SnRK1.2 was constitutively increased in *gpt2*. Furthermore, we saw an increase in the abundance of NMT1 in the WT, which was lacking in *gpt2*. Pierre et al. (2007) have previously shown that the β-SnRK1 subunits are negatively regulated by NMT1, and *nmt1* mutants had enhanced SnRK1 activity. This raises the possibility that SnRK1.2 activity may be suppressed by NMT1 in the WT, whereas this suppression is lacking in *gpt2*. As a result, SnRK1.2 activity may be higher in *gpt2* plants, which is supported by the observation that both *gpt2* and SnRK1.2 overexpressors display an early flowering phenotype relative to the WT (Athanasiou et al., 2010; Williams et al., 2014).

We are not at present able to conclusively define a role for GPT2 in light acclimation. Evidence presented here suggests that there is no simple role – GPT2 is not a sensor controlling the master switch for photosynthetic acclimation. Rather, we suggest that it results in subtle changes across the proteome with different processes being differentially affected by lack of this translocator.

## Conclusion

Analysis of the proteome, using a simple, label-free approach, has allowed us to define in some detail the complex changes occurring in leaves as they acclimate to HL. The consistency of our data with both physiological analysis and previous protein and metabolomic studies gives us confidence in the normalization method and the ability of this technique to unravel intricate responses to the environment. We are able to define key processes in photosynthesis and metabolism which are differentially altered in the *gpt2* mutant and this has allowed us to separate out processes which are separately regulated. We further suggest that this has repercussions for the regulation of metabolism in *gpt2*. Given the non-correlation seen between transcriptomic and functional or proteomic studies, is it clear that the latter will become increasingly important in trying to gain a complete system understanding of metabolism.

## Author Contributions

MM and GJ designed the experiments. MM grew plants, extracted proteins, performed physiological measurements and analyzed data. RO performed mass spectrometry and sample preparation, DK, SH, and JS provided technical guidance for analysis of proteomics. MK carried out analyses of leaf weight and area. All authors contributed to the data analysis. MM and GJ wrote the paper.

## Conflict of Interest Statement

The authors declare that the research was conducted in the absence of any commercial or financial relationships that could be construed as a potential conflict of interest.
