# Supplementary material for: Dynamic Acclimation to High Light in Arabidopsis thaliana Involves Widespread Reengineering of the Leaf Proteome
Source: Front Plant Sci. 2017 Jul 20;8:1239. doi: 10.3389/fpls.2017.01239 (PMC5517461; doi:10.3389/fpls.2017.01239)
Supplement: Supplementary file 4 [file Image_1.PDF]

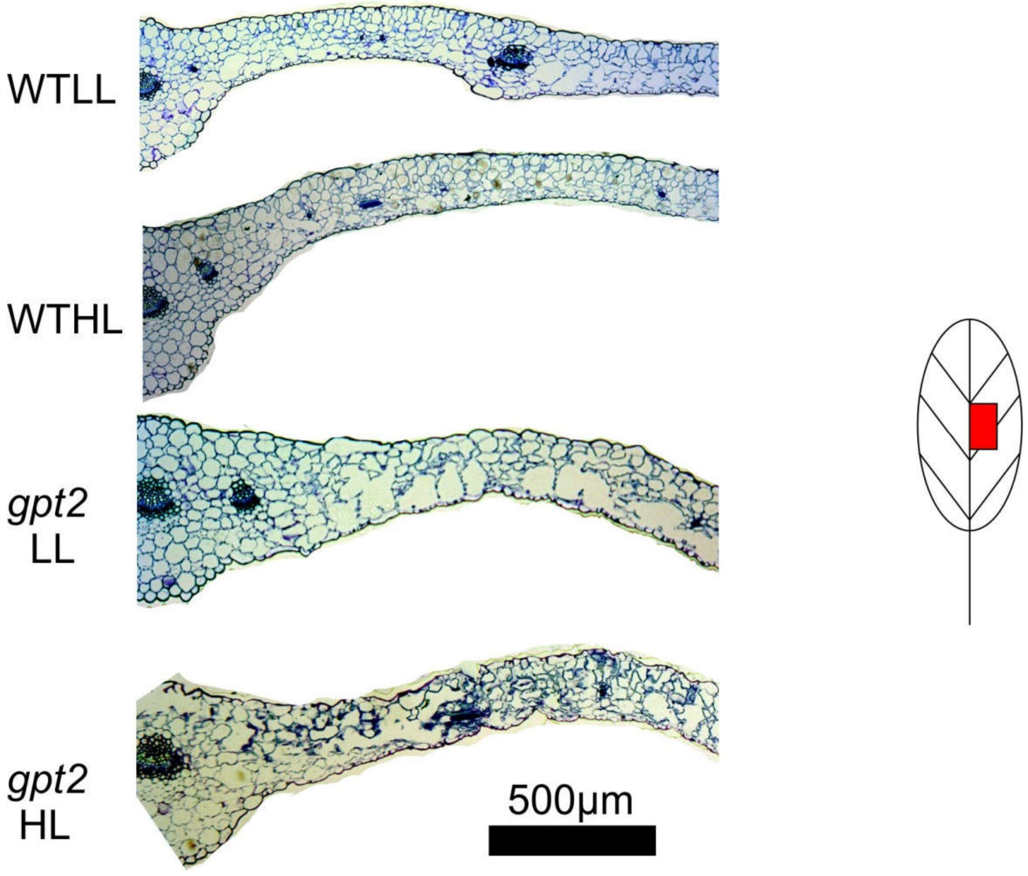

<u>Supplementary Figure S1: Transverse leaf sections of WT and *gpt2* before and after HL acclimation</u>

8-week old WT and *gpt2* leaves maintained at LL (100 µmol m^{-2} s^{-1}) were harvested or acclimated for 7 days at HL (400 µmol m^{-2} s^{-1}) and harvested. Representative images are shown, with no gross changes in leaf morphology being seen during acclimation to HL. Sections of mature leaves, from the midrib extending towards the leaf margin were used, as shown by the red box at the right of the graphic. Harvested leaf sections were dehydrated and embedded in resin using a JB4 embedding kit (Polysciences, Warrington, PA, USA). Sections of 5µm were stained using Toluidine blue, cleared with xylene and mounted using DPX mountant (Sigma).
