# Supplementary material for: Dynamic Acclimation to High Light in Arabidopsis thaliana Involves Widespread Reengineering of the Leaf Proteome
Source: Front Plant Sci. 2017 Jul 20;8:1239. doi: 10.3389/fpls.2017.01239 (PMC5517461; doi:10.3389/fpls.2017.01239)
Supplement: Supplementary file 5 [file Image_2.PDF]

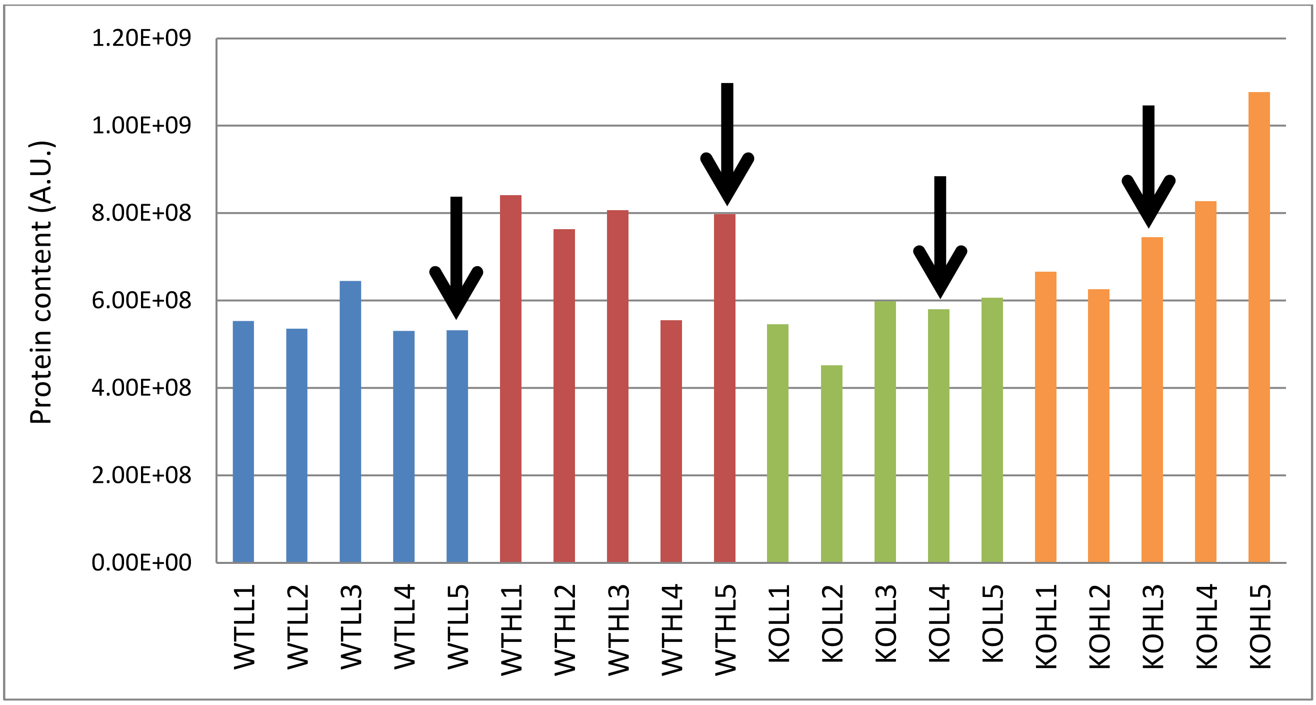

Supplementary Figure S2: Summary of total protein normalisation method

For calculation of total protein content, the intensity of every quantified protein was summed for each sample. Individual samples are named on the x-axis and treatments represented by the colour coded bars, where WT at LL (WTLL) = blue, WT at HL (WTHL) = red, *gpt2* at LL (KOLL) = green and *gpt2* at HL (KOHL) = orange. Reference runs for each treatment were selected by calculating the sample with the median total protein content as indicated by the black arrows. The median total protein content was selected as opposed to the average total protein content to exclude the inclusion of outliers in the calculation (such as WTHL4 and KOHL5). A scaling factor was then calculated for all other samples within the treatment, which was applied to every quantified protein to allow for normalisation to the reference run.
