# Supplementary material for: Dynamic Acclimation to High Light in Arabidopsis thaliana Involves Widespread Reengineering of the Leaf Proteome
Source: Front Plant Sci. 2017 Jul 20;8:1239. doi: 10.3389/fpls.2017.01239 (PMC5517461; doi:10.3389/fpls.2017.01239)
Supplement: Supplementary file 6 [file Image_3.PDF]

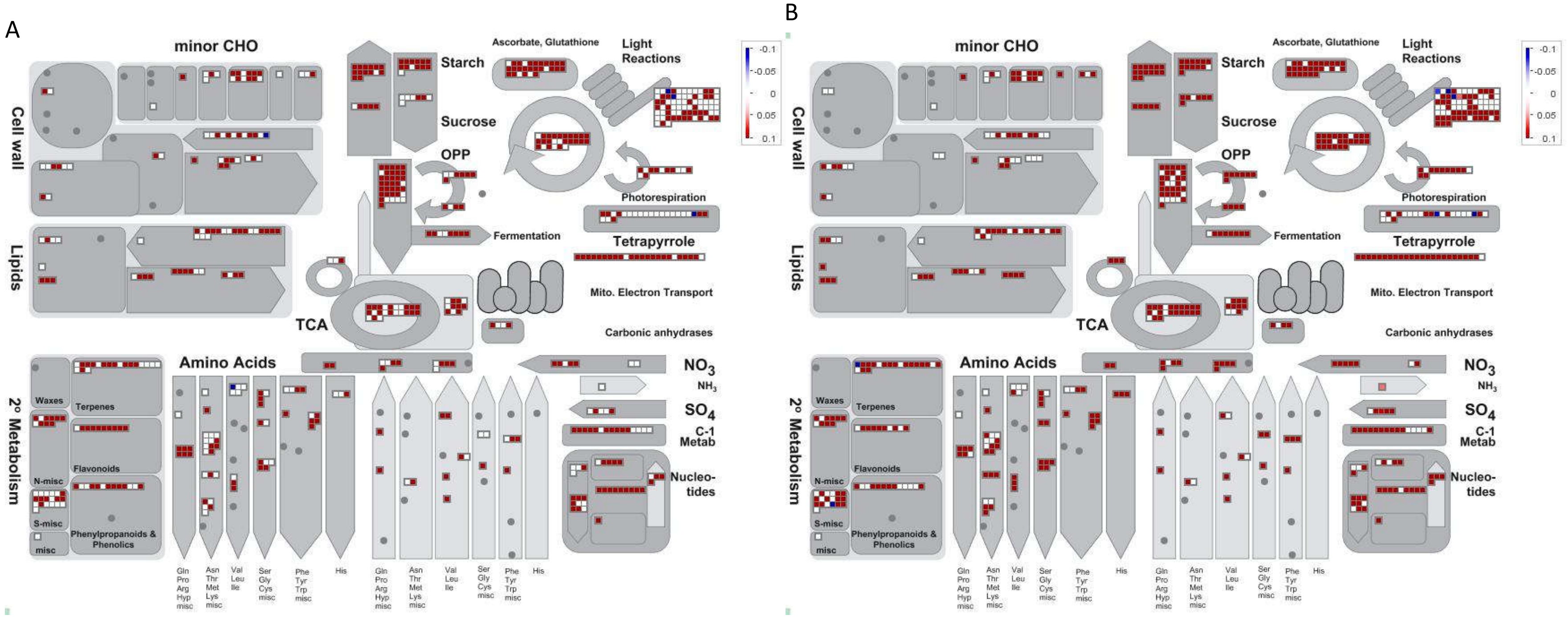

<u>Supplementary Figure S3: Mapman metabolism overview detailing changes in the proteome in the WT (A) and *gpt2*(B)</u>

Fold change data for the 1993 quantified proteins were submitted to Mapman. Red boxes denote proteins that were upregulated, white boxes are proteins that do not significantly change in abundance, and blue boxes are downregulated proteins.
