# Supplementary material for: Dynamic Acclimation to High Light in Arabidopsis thaliana Involves Widespread Reengineering of the Leaf Proteome
Source: Front Plant Sci. 2017 Jul 20;8:1239. doi: 10.3389/fpls.2017.01239 (PMC5517461; doi:10.3389/fpls.2017.01239)
Supplement: Supplementary file 7 [file Image_4.PDF]

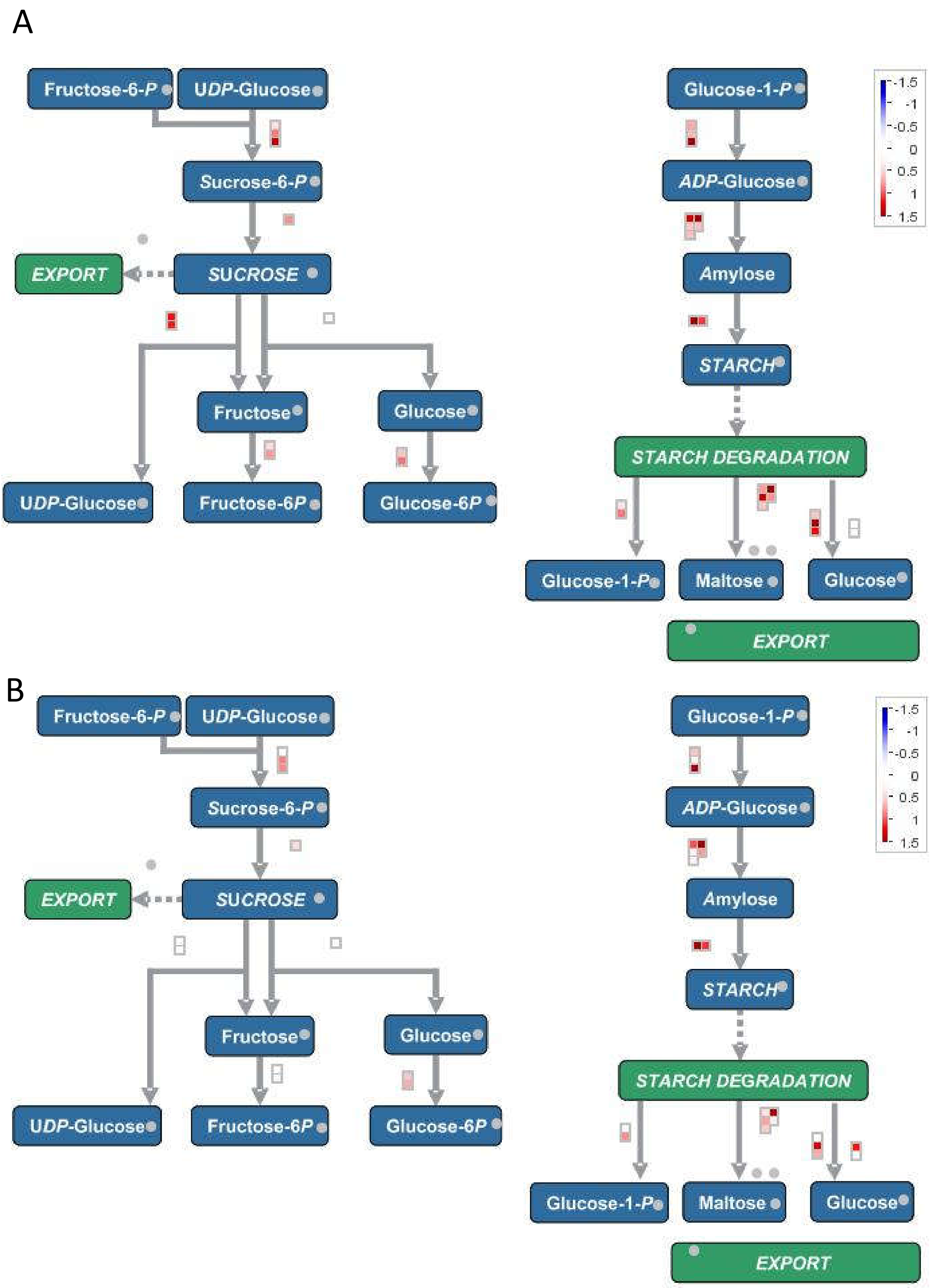

Supplementary Figure S4: Mapman metabolic digram detailing the changes in Sucrose and Starch metabolism after high light acclimation

Fold change data for the WT (A; WTHL/WTLL) and *gpt2* (B; *gpt2*HL/*gpt2*LL) is shown. The major enzymes of sucrose (left) and starch (right) metabolism are shown. Boxes represent induvidual proteins, where red boxes are upregulated, white boxes are non changing and blue boxes are downregulated proteins. More intense colouring indicates a more severe fold change, according to the key to the top right of each figure.
